# Clinical and Hemodynamic Features of Aneurysm Rupture in Coil Embolization of Intracranial Aneurysms

**DOI:** 10.3390/diagnostics14111203

**Published:** 2024-06-06

**Authors:** Tomoaki Suzuki, Hitoshi Hasegawa, Kohei Shibuya, Hidemoto Fujiwara, Makoto Oishi

**Affiliations:** Department of Neurosurgery, Brain Research Institute, Niigata University, 1-757 Asahimachi-Dori, Niigata 951-8585, Japan

**Keywords:** intraprocedural rupture, cerebral aneurysm, coil embolization, computational fluid dynamics, flow impingement zone, pressure, wall shear stress, hemodynamic instability

## Abstract

Intraprocedural rupture (IPR) during coil embolization (CE) of an intracranial aneurysm is a significant clinical concern that necessitates a comprehensive understanding of its clinical and hemodynamic predictors. Between January 2012 and December 2023, 435 saccular cerebral aneurysms were treated with CE at our institution. The inclusion criterion was extravasation or coil protrusion during CE. Postoperative data were used to confirm rupture points, and computational fluid dynamics (CFD) analysis was performed to assess hemodynamic characteristics, focusing on maximum pressure (Pmax) and wall shear stress (WSS). IPR occurred in six aneurysms (1.3%; three ruptured and three unruptured), with a dome size of 4.7 ± 1.8 mm and a D/N ratio of 1.5 ± 0.5. There were four aneurysms in the internal carotid artery (ICA), one in the anterior cerebral artery, and one in the middle cerebral artery. ICA aneurysms were treated using adjunctive techniques (three balloon-assisted, one stent-assisted). Two aneurysms (M1M2 and A1) were treated simply, yet had relatively small and misaligned domes. CFD analysis identified the rupture point as a flow impingement zone with Pmax in five aneurysms (83.3%). Time-averaged WSS was locally reduced around this area (1.3 ± 0.7 [Pa]), significantly lower than the aneurysmal dome (*p* < 0.01). Hemodynamically unstable areas have fragile, thin walls with rupture risk. A microcatheter was inserted along the inflow zone, directed towards the caution area. These findings underscore the importance of identifying hemodynamically unstable areas during CE. Adjunctive techniques should be applied with caution, especially in small aneurysms with axial misalignment, to minimize the rupture risk.

## 1. Introduction

Rupture of cerebral aneurysms, leading to life-threatening subarachnoid hemorrhage (SAH), is a critical concern in cerebrovascular disease. Microsurgical clipping has been the standard treatment for intracranial aneurysms. However, endovascular approaches including coil embolization have become crucial alternatives due to their minimally invasive nature and proven long-term efficacy in preventing aneurysm rupture [1,2,3,4]. Despite advancements in endovascular devices, such as flow diverter stents, coil embolization remains vital for promoting intra-aneurysmal thrombosis and reducing rupture risk. Recent innovations, including the use of flow diverter stents, offer effective solutions for managing challenging aneurysms. However, the risk of mechanical stress on the aneurysmal wall during coil insertion persists, necessitating the careful manipulation of microcatheters and coils. Intra-procedural aneurysm rupture (IPR) is a severe complication of coil embolization with reported incidence rates ranging from approximately 1.4% to 7.7% [5,6,7], often leading to periprocedural mortality or disability [8,9]. The vulnerability of the aneurysm wall to rupture often results from fragile thinning in unruptured aneurysms and the initial rupture point in those that have ruptured, primarily induced by abnormal hemodynamic stress [10,11]. Previous investigations have explored the hemodynamic characteristics of thinning walls in cerebral aneurysms, highlighting the role of abnormal hemodynamic stress, such as static pressure and wall shear stress, in precipitating unstable wall conditions [12,13]. Unlike surgical clipping, endovascular procedures entail inevitable challenges in visualizing aneurysmal wall conditions. An accurate prediction of the location of fragile walls is critical for averting fatal IPR, particularly during endovascular intervention. Despite previous investigations on the hemodynamic characteristics of thinning aneurysm walls [14,15], the specific features of the areas that are at risk of rupture during endovascular treatment remain unexplored. This study aimed to elucidate the clinical and hemodynamic characteristics of IPR in the endovascular management of cerebral aneurysms.

## 2. Materials and Methods

### 2.1. Patients and Aneurysm Characteristics

Between January 2012 and December 2023, 435 patients with intracranial aneurysms (316 unruptured and 119 ruptured) underwent coil embolization at our hospital. We retrospectively investigated patients who experienced extravasation or coil protrusion during coil embolization. The rupture points were identified postoperatively, and computational fluid dynamics (CFD) analysis was performed to assess hemodynamic features. Table 1 summarizes patient characteristics.

### 2.2. Computational Fluid Dynamics Analysis

We used a hemoscope (EBM Corp, Tokyo, Japan) for CFD analysis to study cerebral aneurysm flow dynamics [16], focusing on the pressure and wall shear stress (WSS) at the rupture site. Further details on the analysis are provided below.

### 2.3. Pressure Elevation and Time-Averaged WSS (TAWSS) Were Computed

Vascular geometry was derived from DICOM data from CTA or 3D-DSA images and reconstructed using Ziostation2 software (Ziosoft, Inc., Tokyo, Japan). We employed a pulsatile flow rate in the simulations, solving the Navier–Stokes equations with blood properties modeled as an incompressible Newtonian fluid (density: 1050 kg/m^3^, dynamic viscosity: 0.004 Pa·s). The vessel walls were assumed rigid. The flow rate was calculated based on the uniform wall shear stress hypothesis with the WSS set at 1.5 Pa.

The boundary conditions were established following the constant wall shear stress theory, and the inlet and outlet vessel flow rates were calculated using the following equation [17]:

(1) $${Q=\frac{\tau \pi }{32\mu }D}^{3},$$

where Q, τ, μ, and *D* represent the flow rate, wall shear stress, fluid viscosity, and vascular diameter, respectively. This equation serves as a well-known theoretical basis for fully developed laminar pipe flow. Here, the wall shear stress was fixed at *τ* = 1.5 Pa. The inlet pressure was set to 100 mmHg, and the flow distribution at each outlet was determined in accordance with Murray’s law.

A finite volume method was used to solve the governing equations of the unsteady Navier–Stokes equations and the continuity equation. Blood was assumed to be an incompressible Newtonian fluid, and the blood flow exhibited transient behaviors. The Euler method and a second-order upwind scheme were adopted to discretize the unsteady and convective acceleration terms. The convergent criteria were set at 10^−4^.

### 2.4. Statistical Analysis

An unpaired two-sample *t*-test was used to compare baseline characteristics between groups. The statistical significance was set at a nominal *p*-value <0.05. All statistical analyses were performed using GraphPad Prism version 7.02 (GraphPad Software, San Diego, CA, USA).

## 3. Results

**Figure 1:**
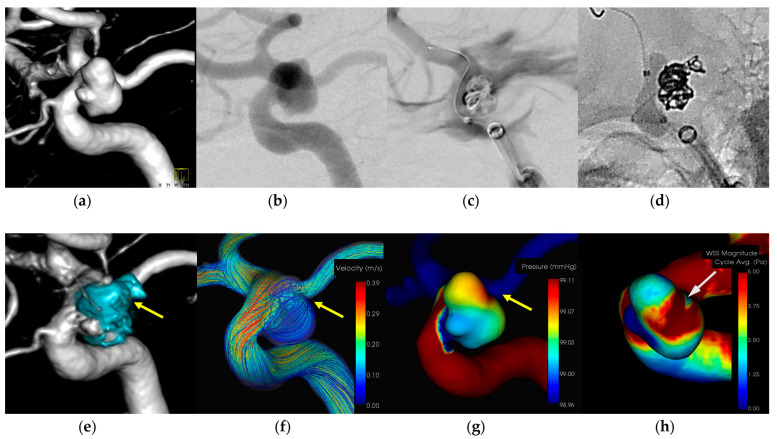
In Case 1, an unruptured right internal carotid paraclinoid aneurysm (dome size: 6.5 mm) was treated by balloon-assisted coiling. (**a**) Volume rendering (VR) image (pretreatment). (**b**) Digital subtraction angiography (DSA) image (pretreatment). (**c**) Extravasation after inserting the framing coil. (**d**) Coil protrusion from intraprocedural rupture (IPR) site (yellow arrow). (**e**) VR image (posttreatment) and coil protrusion from IPR site (yellow arrow). (**f**) The IPR site corresponds to the flow impingement zone (yellow arrow). (**g**) The IPR site corresponds to the maximum pressure area (yellow arrow). (**h**) Wall shear stress decreased locally around the IPR site (white arrow: TAWSS 0.9 [Pa]).

**Figure 2:**
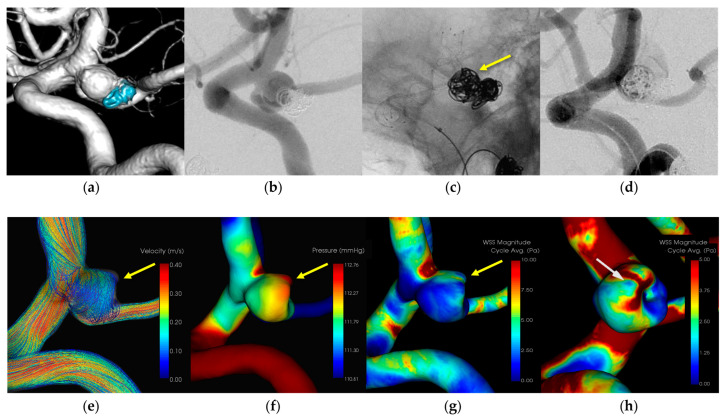
In Case 2, an unruptured recurrent left internal carotid—posterior communicating artery aneurysm (dome size: 5.0 mm) was treated by stent-assisted coiling. (**a**) Volume rendering (VR) image (pretreatment). (**b**) Digital subtraction angiography (DSA) image (pretreatment). (**c**) Stent-assisted coiling with LVIS and the head of the microcatheter (yellow arrow). (**d**) Extravasation after inserting the filling coil and the rupture point of the aneurysmal dome around the head of the microcatheter. (**e**) The IPR site corresponds to the flow impingement zone (yellow arrow). (**f**) The IPR site corresponds to the maximum pressure area (yellow arrow). (**g**) Wall shear stress decreased locally around the IPR site (yellow arrow). (**h**) Localized low TAWSS around the IPR site is 1.5 [Pa] (white arrow).

**Figure 3:**
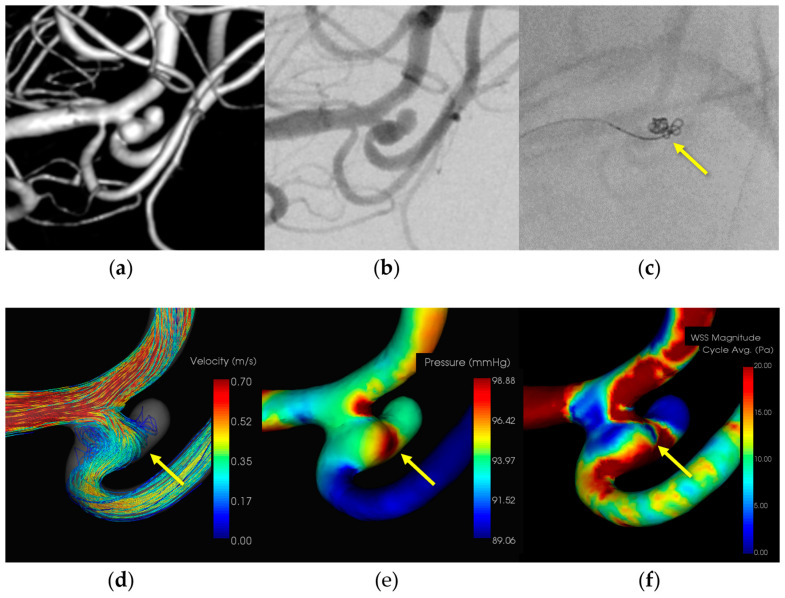
In Case 3, a ruptured left middle cerebral artery aneurysm (dome size: 4.0 mm) was treated by simple coiling. (**a**) Volume rendering (VR) image (pretreatment). (**b**) Digital subtraction angiography (DSA) image (pretreatment). (**c**) Coil protrusion from intraprocedural rupture (IPR) site (yellow arrow). (**d**) The IPR site corresponds to the flow impingement zone (yellow arrow). (**e**) The IPR site corresponds to the maximum pressure area (yellow arrow). (**f**) Wall shear stress decreased locally around the IPR site (yellow arrow: TAWSS 2.3 [Pa]).

**Figure 4:**
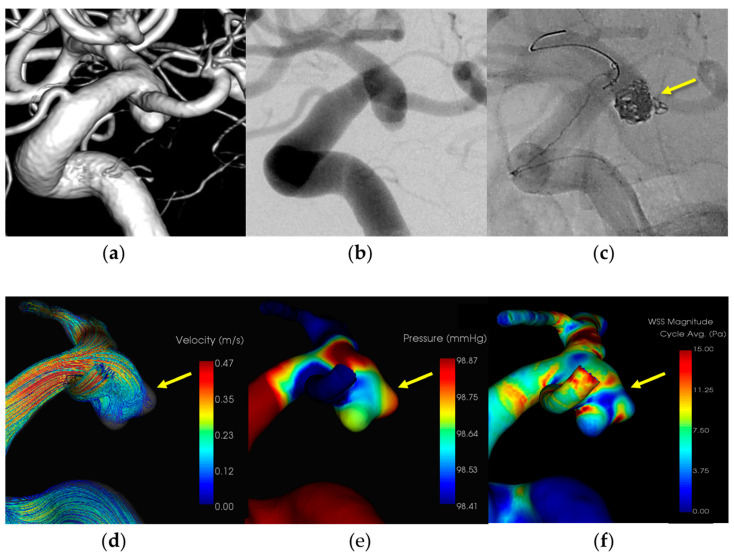
In Case 4, a ruptured left internal carotid—posterior communicating artery aneurysm (dome size: 6.8 mm) was treated by balloon-assisted coiling. (**a**) Volume rendering (VR) image (pretreatment). (**b**) Digital subtraction angiography (DSA) image (pretreatment). (**c**) Coil protrusion from intraprocedural rupture (IPR) site (yellow arrow). (**d**) The IPR site corresponds to the flow impingement zone (yellow arrow). (**e**) The IPR site corresponds to the maximum pressure area (yellow arrow). (**f**) Wall shear stress decreased locally around the IPR site (yellow arrow: TAWSS 1.6 [Pa]).

During coil embolization, rupture occurred in six aneurysms (1.3%). These included four internal carotid artery (ICA) aneurysms, one anterior cerebral artery aneurysm, and one middle cerebral artery aneurysm. All ICA aneurysms were treated with an adjunctive technique (three balloon-assisted and one stent-assisted technique), whereas the remaining two aneurysms (M1M2 and A1) were treated using a simple technique. Notably, the aneurysm dome in these cases was relatively small and axially misaligned from the bifurcation. The site of rupture was identifiable in five cases (83.3%), with a noted increase in pressure consistent with the blood flow collision site (Pmax area). The mean time-averaged WSS at the rupture sites was 1.3 ± 0.7 Pa, which was significantly lower than that of the aneurysmal dome (*p* < 0.01); this indicates a correlation between low WSS and rupture risk. During coil embolization, the microcatheter tip was positioned along the inflow zone and directed toward the flow impingement areas. Table 2 presents the results of the CFD analysis, and the representative cases are shown as Figure 1, Figure 2, Figure 3 and Figure 4.

### 3.1. Representative Cases

#### 3.1.1. Case 1 (Figure 1)

- Patient: Female, 40s, unruptured right ICA aneurysm.
- Technique: Balloon-assisted coiling.
- Findings: Extravasation after initial coiling, hemostasis achieved with balloon inflation and additional coiling.
- Outcome: Discharge without neurological deficits.
- CFD Results: Pressure elevation and low WSS at the rupture site.

A female patient in her 40s with an unruptured right ICA aneurysm underwent balloon-assisted coiling. Extravasation occurred during the procedure and hemostasis was achieved by inflating the balloon and adding coils. The patient was discharged without neurological deficits. CFD analysis indicated pressure elevation and low WSS at the rupture site. Figure 1 illustrates the hemodynamic characteristics observed in Case 1, showing the pressure distribution and WSS around the rupture site.

An 8-Fr Roadmaster catheter (Goodman, Aichi, Japan) was used as the guiding catheter, and coil embolization was performed using a Phenom 17 microcatheter (Medtronic Neurovascular, Irvine, CA, USA) coupled with a 072 Navien intermediate catheter. A SHOURYU HR (Kaneka Medics, Kanagawa, Japan) 4 × 7 mm balloon catheter was deployed at the aneurysmal neck for balloon-assisted coiling. Following the initial frame coiling without coil protrusion, a contrast medium injection revealed extravasation. The balloon was then inflated to achieve hemostasis, resulting in the protrusion of an additional coil from the region around the head of the microcatheter. The subsequent insertion of additional coils promptly halted bleeding. CFD analysis indicated the location of the IPR at the coil protrusion site, suggesting pressure elevation with Pmax due to flow impingement. A low WSS was localized around the rupture area.

#### 3.1.2. Case 2 (Figure 2)

- Patient: Female, 70s, unruptured left ICA aneurysm.
- Technique: Stent-assisted coiling.
- Findings: Extravasation during coil embolization, hemostasis achieved with additional coiling.
- Outcome: Discharge without neurological deficits.
- CFD Results: Pressure elevation and low WSS at the rupture site.

A female patient in her 70s who had undergone coil embolization for an unruptured left internal carotid artery—posterior communicating artery aneurysm 12 years prior underwent retreatment with stent-assisted coil embolization. Extravasation occurred during coil embolization and hemostasis was achieved by adding coils. The patient was discharged without neurological deficits. CFD analysis indicated pressure elevation and low WSS at the rupture site. Figure 2 illustrates the hemodynamic characteristics observed in Case 2, showing the pressure distribution and WSS around the rupture site.

A 7-Fr Enboy catheter (Codman Neuro, Raynham, MA, USA) served as the guiding catheter in the internal carotid artery, while a RESTAR microcatheter (Medico’s Hirata, Osaka, Japan) was positioned within the aneurysm. Stent placement was facilitated using a Headway 21 microcatheter (Terumo Microvention, Tokyo, Japan). Coil embolization was performed following LVIS deployment (Microvention, Aliso Viejo, CA, USA). Extravasation was observed during coil filling and originated from the rupture point of the aneurysmal dome around the microcatheter head. Additional immediate coil embolization effectively prevented bleeding. CFD analysis identified the extravasation site as the area of the IPR, suggesting pressure elevation with Pmax due to flow impingement. A localized low WSS was also identified around the rupture area.

#### 3.1.3. Case 3 (Figure 3)

- Patient: Female, 50s, ruptured left MCA M1–M2 bifurcation aneurysm.
- Technique: Simple coiling.
- Findings: Extravasation after initial coiling, hemostasis achieved with balloon inflation and additional coiling.
- Outcome: Discharge without neurological deficits due to IPR.
- CFD Results: Pressure elevation and low WSS at the rupture site.

A female patient in her 50s with a subarachnoid hemorrhage (Hunt and Kosnik grade 3) resulting from a ruptured left MCA M1-M2 bifurcation aneurysm underwent simple coiling. Extravasation occurred during the procedure and hemostasis was achieved by adding coils. The patient was discharged without neurological deficits. CFD analysis indicated pressure elevation and low WSS at the rupture site. Figure 3 illustrates the hemodynamic characteristics observed in Case 3, showing the pressure distribution and WSS around the rupture site.

An 8-Fr Roadmaster catheter (Goodman, Aichi, Japan) was positioned in the internal carotid artery as a guiding catheter, while coil embolization was conducted using a Phenom 17 microcatheter paired with a Cerulean DD6 intermediate catheter. During coil filling, coil protrusion from the aneurysm dome and extravasation from the rupture point in the middle of the aneurysm dome were observed. Immediate additional coil embolization successfully halted bleeding. Subsequently, the patient was transferred to a rehabilitation hospital because of the initial damage caused by the subarachnoid hemorrhage. CFD analysis identified the location of the IPR at the site of extravasation, indicating pressure elevation with Pmax attributed to flow impingement. Additionally, there was a localized decrease in the WSS in the ruptured area.

#### 3.1.4. Case 4 (Figure 4)

- Patient: Female, 70s, ruptured left ICA aneurysm.
- Technique: Balloon-assisted coiling.
- Findings: Coil protruded from the aneurysm dome during coiling, hemostasis was achieved with balloon inflation, and additional coiling.
- Outcome: Discharge without neurological deficits.
- CFD Results: Pressure elevation and low WSS at the rupture site.

A female patient in her 70s had a subarachnoid hemorrhage (Hunt and Kosnik grade 3) resulting from a ruptured left internal carotid artery—posterior communicating artery aneurysm, which was managed with a balloon-assisted coil. Extravasation occurred during the procedure and hemostasis was achieved by adding coils. The patient was discharged without neurological deficits. CFD analysis indicated pressure elevation and low WSS at the rupture site. Figure 4 illustrates the hemodynamic characteristics observed in Case 4, showing the pressure distribution and WSS around the rupture site.

An 8-Fr Roadmaster catheter (Goodman, Aichi, Japan) was positioned in the internal carotid artery as a guiding catheter and coil embolization was performed using an Excelsior SL-10 microcatheter (Boston Scientific, Natick, MA, USA) paired with a Cerulean DD6 intermediate catheter. A SHOURYU HR (Kaneka Medics, Kanagawa, Japan) 4 × 7 mm balloon catheter was deployed at the aneurysmal neck for balloon-assisted coiling. During coil filling, protrusions of the coils from the aneurysm dome were observed. Immediate additional coil embolization successfully halted bleeding. CFD analysis identified the location of the IPR at the site of extravasation, indicating pressure elevation with Pmax attributed to flow impingement. Additionally, there was a localized decrease in the WSS in the ruptured area.

## 4. Discussion

IPR is a major concern for endovascular surgeons because of its implications in mortality and morbidity [9,18]. Our study highlights specific hemodynamic characteristics such as high pressure and low WSS at rupture sites. While the use of endovascular techniques has surged along with the development of devices such as coils, microcatheters, and stents for cerebral aneurysm treatment, the risk of IPR persists, even with meticulous manipulation. Unlike open surgery, in which the thinning of the aneurysmal wall signals rupture susceptibility under passive mechanical stress from the coils, the condition of the aneurysmal wall remains elusive during endovascular procedures. Flow impingement and pressure elevation are significant risk factors for cerebral aneurysm rupture, and are closely linked to hemodynamic instability. In these zones, the microcatheter tends to navigate and the inserted coil has the potential to exert pressure on vulnerable walls along the inflow angle. This study identified areas of high pressure and low WSS at the rupture sites, suggesting that these hemodynamic factors significantly contribute to the risk of IPR. A low WSS was noted to cause endothelial cell dysfunction and wall degeneration, contributing to aneurysm fragility. CFD has emerged as a crucial tool for identifying vulnerable areas prone to rupture during coil embolization. By highlighting the zones of high pressure and low WSS, CFD provides valuable insights into the hemodynamic stresses that contribute to aneurysm rupture.

### 4.1. Hemodynamic Findings and Aneurysmal Wall Condition with IPR

Previous studies have demonstrated the role of hemodynamic stress in aneurysm rupture [19,20]. Flow impingement and pressure elevation are significant risk factors of cerebral aneurysm rupture. Our findings corroborate those of these studies, revealing that flow impingement zones with elevated pressure and low WSS are critical for predicting IPR. In this study, we detected this hemodynamic feature at the intraoperative rupture point in most IPR aneurysms (one ICA aneurysm showed no Pmax area at the rupture site; however, it was adjacent to the Pmax area). Previous CFD studies, in conjunction with operative observations during clipping, have revealed that thinning wall areas exhibit the highest pressure with flow impingement in unruptured aneurysms [13,21]. Such hemodynamic stress is often concentrated in the aneurysmal wall surrounding the inflow zone traversed by the microcatheter during coil embolization, indicating the possible presence of thin and fragile aneurysmal walls in this region. Flow impingement, as described in prior CFD studies, is recognized as a hazardous hemodynamic feature predisposing to aneurysmal rupture [22,23]. Furthermore, excessive hemodynamic stress accompanied by pressure elevation is believed to initiate the thinning and fragility of rupture-prone walls [24,25], potentially resulting in severe bleeding. WSS represents another critical hemodynamic stress factor strongly associated with aneurysm growth and rupture [26,27]. Particularly, low WSS has been robustly linked to aneurysm rupture, with previous research indicating its role in endothelial cell dysfunction and wall degeneration within aneurysms [28,29]. In our study, localized low WSS was observed around the IPR site, which notably decreased within the aneurysmal dome. These findings suggest that a low WSS also contributes significantly to the hemodynamic stresses underlying IPR, possibly due to the presence of fragile aneurysm walls.

### 4.2. Mechanical and Hemodymanic Stress on Aneurysm Wall with IPR

The mechanical stress induced by the coils is another significant factor in IPR. This stress poses the risk of stiffness and friction within the aneurysmal wall, potentially leading to rupture during coil embolization, particularly in cases of small aneurysms or when the microcatheter exhibits reduced flexibility [30]. Two cases of IPR (A1 and M2) were managed using a straightforward technique with relatively small dome sizes and axial misalignment from the bifurcation, which possibly resulted in microcatheter fixation. Morphologically, small aneurysms have been identified as a risk factor for IPR [31]. Adjunctive techniques such as balloon- and stent-assisted procedures have the potential to induce IPR due to microcatheter fixation during coil insertion [32]. In our investigation, all four ICA IPR cases were treated with adjunctive techniques. Extravasation occurs during coil embolization, leading to a gradual loss of microcatheter mobility. The trajectory of the microcatheter followed the inflow streamline into the aneurysm, culminating in an impingement zone at the site of intraoperative rupture. The use of adjunctive techniques warrants careful consideration because of the risk of excessive stress on the aneurysm wall, particularly within the flow impingement zone where the wall may be particularly fragile. A combination of CFD analyses for the IPR revealed that the lower flexibility of the microcatheter could increase the mechanical stress during coil insertion in the flow impingement zone, leading to pressure elevation and low WSS, indicating potential fragile thinning or ruptured walls. Notably, no prior studies have explored the CFD analysis of the IPR site in cerebral aneurysms.

Although endovascular treatment has increasingly adopted a flow-diversion strategy, coil embolization remains indispensable for preventing aneurysm rupture. Unlike microsurgical clipping, endovascular surgeons lack direct visibility of the aneurysmal wall, necessitating caution regarding contact during the procedure. To our knowledge, this is the first study to identify specific hemodynamic characteristics such as high pressure and low WSS at rupture sites during coil embolization, underscoring the elevated risk of IPR in these areas. Our findings may serve as a foundation for employing CFD analyses to mitigate catastrophic IPR. The identification of hemodynamic risk factors, such as high pressure and low WSS, can inform endovascular strategies to minimize the risk of IPR. Surgeons should exercise caution when navigating microcatheters through flow impingement zones and should consider the potential benefits of CFD analysis in preoperative planning.

### 4.3. Limitations

This study has a few limitations. The small sample size limits the generalizability of our findings and necessitates larger studies for validation. Additionally, the CFD analysis did not incorporate patient-specific boundary conditions, which could enhance accuracy if obtained through advanced imaging techniques. Future research should aim to integrate these factors and explore their implications in the open surgery setting.

## 5. Conclusions

Our study confirmed that the aneurysm walls are prone to thinning at sites of pressure elevation due to blood flow impingement, which significantly increases the risk of IPR. Utilizing hemoscope-based CFD analysis to predict regions at risk of rupture can enhance clinical decision-making and improve patient outcomes by allowing targeted interventions to prevent fatal IPR. The study’s findings highlight the critical role of CFD analysis in identifying the hemodynamic risk factors for IPR, providing a valuable tool for enhancing the safety and efficacy of endovascular treatment for cerebral aneurysms.

## Figures and Tables

**Table 1:** Patient characteristics of IPR.

*N* = 6	
Sex (female:male)	5:1
Mean age (years)	62.8 ± 15.3
Aneurysm Size	
Dome size (mm)	4.7 ± 1.8
Neck size (mm)	3.2 ± 0.8
D/N	1.5 ± 0.5
Aneurysm Location	
ICA	4
MCA	1
ACA	1
Treatment	
Simple coiling	2
Ballon-assisted coiling	3
Stent-assisted coiling	1

**Table 2:** Hemodynamic characteristics of the patients with IPR.

**Flow impingement zone (Pmax) = IPR site**	5/6 (83.3%)
**Pressure**	
IPR site (mmHg)	109.4 ± 6.4
**TAWSS**	
IPR site (Pa)	* 1.3 ± 0.7
Dome (Pa)	5.1 ± 3.7

* significantly lower than TAWSS of the aneurysmal dome (*p* < 0.01).

## Data Availability

The original contributions presented in the study are included in the article, and further inquiries can be directed to the corresponding author.
